# Global health without justice or ethics

**DOI:** 10.1093/pubmed/fdaa001

**Published:** 2020-04-06

**Authors:** S Venkatapuram

**Affiliations:** King’s Global Health Institute, King’s College London, London, SE1 9NH, UK

The great promise at the start of the twenty-first century that Anglo-American philosophers would produce transformative theories and practical guidance for realizing global health equity and justice has largely gone unfulfilled. The publication of *The Law of Peoples* by John Rawls in 1999 formally inaugurated the emerging academic field of global justice philosophy.^1^ After 2000, numerous monographs, journal articles and conferences discussed global justice. And new academic associations, journals and research centres were established.

One remarkable aspect of the new field was that the stark inequalities in health across societies were often the starting concern. Despite our diverse philosophical and ethical views, reasonable people are likely to be morally troubled about the large inequalities in life expectancies between some sub-Saharan country X and the USA or another rich country. This initially shared moral intuition or indignation, then, motivated diverse arguments about what precisely is morally bad about global health inequalities and global poverty and the possible demands of justice. Some philosophers described what ‘our’ duties are or, indeed, are not, to help ‘those people over there’. Others minimized the distinction between us and them by arguing for theories of radical global equality, the arbitrariness of political borders and duties that follow from our complicity in transnational harms experienced in other countries.

Progress in global justice philosophy seemingly promised real-world progress in global health equity and justice, because health inequality was the foremost issue in philosophical debates on global inequality, poverty and claims of the ‘global poor’. At the same time, largely driven by HIV research, bioethics went global as it was exported alongside medical research to resource poor settings. Bioethicists also began to go beyond clinical and research settings to examine public health ethics, social inequalities in health and social determinants—from local conditions all the way to global institutions and processes. Nevertheless, as of 2020, it is difficult to identify any compelling conceptions of global justice or global health justice or to identify any significant philosophical contributions to the practical improvement of global health and inequalities. What happened?

This unfulfilled promise and potential of global justice philosophy hang like the bunch of sour grapes of Aesop’s fable against the background of a collapsing international order, questionable ethical practices of global institutions and their leaders and countries blatantly violating international agreements and norms that were thought inviolable. Global health has not been protected from these events. A clear illustration is the attacks on Ebola health centres in the Democratic Republic of Congo (DRC) and the killing of a WHO doctor on 19 April 2019. Attacks persist because of the lack of trust between communities and health workers and international organizations, as well as the continued global toleration of the drivers of the armed conflict. At the same time, an unprecedented 65 million displaced people are living daily lives of uncertainty and vulnerability, many needing physical and mental health care. Health crises with global dimensions are also happening in Syria, South Sudan, Yemen, Central African Republic, Myanmar, Venezuela, Libya, Puerto Rico and more.

New and persistent health crises in different parts of the world as well as widening health inequalities within countries are not due to lack of resources or biomedical knowledge. I would argue that the singular failure of philosophers and global health policy planners and practitioners has been our failure to create and engender moral motivation, a will—among those who are able—to prevent millions of human deaths and create conditions for good health within and across countries. The major global health actors, including funders, do not even share a minimal concept of or commitment to equity.

Rawls stated that given diverse and reasonable philosophical and religious conceptions, one role of political philosophy is to push the limits of what is practically possible in designing political and social institutions such that individuals are viewed and act as free, equal and normal cooperating members over a complete life, across generations.^2^ Pushing the limits meant understanding what currently is the real world and stretching or pulling that towards the best social order that can be imagined, the best world we can hope for. Now, more than ever, we need philosophers like Rawls who can imagine realistic utopias in global health and beyond.
